# The Role of Soluble Polysaccharides in Tannin-Cell Wall Interactions in Model Solutions and in Wines

**DOI:** 10.3390/biom10010036

**Published:** 2019-12-25

**Authors:** Andrea Osete-Alcaraz, Ana Belén Bautista-Ortín, Encarna Gómez-Plaza

**Affiliations:** Department of Food Science and Technology, Faculty of Veterinary Science, University of Murcia, Campus de Espinardo, 30100 Murcia, Spain

**Keywords:** proanthocyanidins, tannins, pectin, mannan, cell walls, polysaccharides, wines, color

## Abstract

The interactions between tannins and soluble and insoluble cell wall components are, in part, responsible for the low quantities of tannins found in wines compared with the quantities in grapes. The use of polysaccharides to compete with cell wall components could be an interesting approach for improving the chromatic and sensory characteristics of wines. The effect of two commercial polysaccharides, pectin and mannan, on limiting tannin-cell wall interactions was studied in a model solution, measuring the concentration of tannins and polysaccharides remaining in solution after the different interactions by chromatography. The treatment was also tested in a small-scale vinification. Soluble polysaccharides were added to the must and the wines were evaluated at the end of alcoholic fermentation and after six months in the bottle. In the model solution, the commercial polysaccharides formed soluble complexes with the tannins and limited the interactions with cell wall components, with some differences between skin and seed tannins. In the case of the wines, the treatments resulted in wines with a higher color intensity and phenolic content. Sensory analysis resulted in higher scores for the wines with added polysaccharides, since the complexation of tannins with the polysaccharides increased the roundness and body of the resulting wines.

## 1. Introduction

The phenolic composition of wines determines their quality. Compounds such as proanthocyanidins, commonly known as tannins, provide important organoleptic characteristics to wines, including body and color stability, although they may also contribute to astringency and dryness sensations. These compounds are extracted from the grape skin and seeds into must-wine during the maceration.

The final concentration of phenolic compounds in the must/wines is usually lower than what might be expected given the concentration measured in grapes. The reason for this is that, during the first stages of vinification, the large amount of vegetal material/cell walls in suspension arising from the degradation of the grape skin and flesh interacts and binds the extracted tannins [1,2]. In previous studies, our research group found that removing some of this suspended vegetal material before the skin maceration stage improved the final wine chromatic characteristics due to the increase of the phenolic content, especially in the concentration of tannins [3].

Cell wall structural polysaccharides interact with phenolic compounds through well-known mechanisms that have been studied in grapes and other plants [4,5,6]. Hydrophobic interactions and hydrogen bonds have been shown to drive the main associations that occur between tannins and cell wall polysaccharides, while the strength of such interactions depends on the structure and conformation of both the tannins and the cell wall materials [7,8]. For example, Ruiz-García et al. [9] showed that the pectic fraction of the cell walls is the fraction with the highest binding capacity for tannins and that, by eliminating this fraction from the cell walls with a chelating agent, the interaction with tannins could be significantly reduced. They also determined that, besides pectic polysaccharides, the hemicellulose present in cell walls also has a high binding capacity, although lower than that observed when pectin is present.

Therefore, one way to limit the interactions between cell walls and tannins, and thus avoid their loss during vinification, could be the use of maceration enzymes, which are, generally, a cocktail of pectolytic, cellulase, and hemicellulase enzymes. They are used with the objective of breaking down the structure of the cell walls to allow the extraction of the phenolic compounds located inside the cells but they could also contribute to deconstructing the polysaccharide network of the cell walls that remain in suspension during the maceration period [10,11], thus reducing their capacity to absorb tannins. Several studies have been carried out with the objective of increasing our knowledge of the role of maceration enzymes in tannin-cell wall interactions [2,5,12,13,14], and it has been commonly observed that the effect of enzymes in increasing the tannin concentration in the medium is limited. Bindon et al. [2], in a study to explain this low effect, observed that, as expected, the cell walls previously treated with a commercial pectolytic enzyme (polygalacturonase) decreased their ability to interact with tannins in model solutions. However, the problem was that, after the enzymatic treatment, if the solubilized part of the cell walls was left in contact with tannins, precipitates were observed. So, despite the use of maceration enzymes, precipitation might still occur, along with the loss of tannins, this time due to reactions between the solubilized material of the cell walls and the tannins.

In a search for a possible solution to this problem, we turned our attention to a previous study by Renard et al. [5] in which adsorption tests between apple cell walls and tannins were carried out in model solution. In an attempt to desorb the tannins linked to the polysaccharide cell walls, the author washed the complexes with model solutions to which soluble polysaccharides had been added, observing higher tannin release than in the absence of polysaccharides, probably due to competition between cell wall-linked polysaccharides and those soluble in the medium.

The use of polysaccharides in enology has long been a common practice. It is known that they can limit the formation of insoluble tannin–protein complexes [15], have a positive impact on wine mouthfeel [16], and increase polyphenol stabilization [17]. As an example, mannoproteins have traditionally been used in finished wines to stabilize wine tannins although contradictory results can be found in the literature [18,19], probably due to the presence of a protein fraction in the mannoprotein molecule.

Based on all the above observations, the main objectives of this work were twofold: (a) to study the addition of two different commercial soluble polysaccharides to a model solution is able to limit the interactions that occur between tannins and grape cell walls through a competitive reaction and (b) to study, in real red wine vinifications, the effect of adding these two polysaccharides to the must, as opposed to finished wines, to see whether the phenolic content and chromatic and organoleptic characteristics of the wines are improved.

## 2. Materials and Methods 

### 2.1. Grape Samples

*Vitis vinifera* L. cv. Monastrell red grapes were harvested at optimum maturity in 2017 from a commercial vineyard located in Jumilla, Murcia (Spain). The grapes were used to isolate the cell walls for the different experiments and to perform the different microvinifications. The grapes used in the microvinifications were stored refrigerated (3 °C) prior to the winemaking process started, and those used to isolate the cell walls for the model solution tests were stored frozen (−18 °C).

### 2.2. Extraction of the Cell Walls from the Grape Skin

Cell walls (CW) were isolated following the method described by De Vries et al. [20] and adapted by Ruiz-García et al. [9].

Fresh skins of *Vitis vinifera* L. Monastrell were used to extract purified cell walls. Initially, a scalpel was used to separate the skin from the other parts of the frozen grapes. Skins were washed with miliQ water and were frozen using liquid N_2_ (−196 °C). Frozen skins were crushed manually, suspended in boiled miliQ water for 5 min, and homogenized at 10,000 rpm for 1 min using a PRO D-Series benchtop homogenizer (PRO Scientific, Oxford, MS, USA). The homogenized skins were centrifuged for another 10 min at 18,000× *g* and the supernatant was discarded. Part of the homogenized material was mixed (1:2) with ethanol (70% v/v) and heated on a hot plate (50 °C) for 30 min while being stirred (5000 rpm). The mixture was then centrifuged for 10 min at 18,000× *g*. A solved sugar evaluation was made, proposed by Dubois et al. [21]. Washing with ethanol (70% v/v) was repeated until the sugar disappeared from the ethanolic phase. Finally, the solids insoluble in alcohol (SIA) were washed once with alcohol (96% v/v), and once with acetone (100% v/v), before being dried under an air flow (25 °C) and stored in darkness. The cell wall polysaccharide profile can be found in [22].

### 2.3. Tannins Used in the Model Solution Experiements

A seed-derived tannin (TanReactive, Agrovin, Alcazar de San Juan, Spain) and a skin-derived commercial tannin (TanSutil, Agrovin, Alcazar de San Juan, Spain) were used in the experiment. The seed-derived tannin had a conversion factor of 71.34% and the skin-derived tannin of 35.41%.

### 2.4. Soluble Polysaccharides Used in Model Solution Experiments and Microvinifications.

Two commercial polysaccharides were used for the experiments: high methylated pectin from citrus fruit (PEC, Sigma Aldrich, ref. no. P-9561, Darmstadt, Germany) and mannan extracted from *Saccharomyces cerevisiae* (MAN, Sigma Aldrich ref. no. M7504, Darmstadt, Germany). The calculated molecular weights were 55,731.74 uma for PEC and 30,492.41 uma for MAN.

In the case of the model solutions, another type of polysaccharides was used in the experiments. These were isolated from the soluble material obtained when cell walls were stirred in a model solution (CW-PS). To obtain this fraction, the CW were suspended in the model solution and stirred for 90 min at 300 rpm, after which the supernatant containing the solubilized material was collected, centrifuged (18,000× *g* for 5 min), and concentrated.

### 2.5. Interaction Test with Tannins, Soluble Polysaccharides, and Cell Walls

Initially, an interaction test was carried out between the two tannins (individually) and the soluble polysaccharides: esterified pectin (PEC) and mannan (MAN). The tannins were initially dissolved in 2.5 mL of a solution with 12% ethanol and pH 3.6 adjusted with trifluoroacetic acid (model solution) and introduced into 3 mL plastic tubes, presenting a final concentration of 2 mg/mL. Subsequently, the soluble polysaccharides were added (separately). The PEC was used at a final concentration of 100 mg/L and MAN was used at a final concentration of 8.3 mg/L. Next, tubes were shaken first in a vortex and then in an orbital shaker for 90 min (25 °C). After this interaction time, the samples were centrifuged at 18,000× *g* for 5 min and the supernatant was concentrated and dried under vacuum at 35 °C in a vacuum CentriVap (Vacuum concentrator, Labconco, MO, USA).

For interactions with the CW-PS, the concentrated material was dissolved in 2.5 mL of the model solution in which the commercial tannin (skin/seed) had previously been dissolved and another interaction test was performed (same conditions and concentrations as in the previous ones).

For interactions in the presence of cell walls, skin cell walls were introduced in 3 mL plastic tubes and mixed with two enological tannins from skin and seed (used separately) that were dissolved previously in 2.5 mL of a solution with 12% ethanol and at pH 3.6 adjusted with trifluoroacetic acid (model solution). The final volume was 2.5 mL, the concentration of the cell walls was 13 mg/mL, and the concentration of the tannins was 2 mg/mL. Next, the soluble polysaccharides were added, except for the control samples (CW + TanSeed; CW + TanSkin) to which no soluble polysaccharide was added. To the other samples, the two different polysaccharides were added: PEC (100 mg/L) and MAN (8.3 mg/L). Tubes were then shaken, first in a vortex and then in an orbital shaker for 90 min (25 °C). After this interaction time, the samples were centrifuged at 18,000× *g* for 5 min and the supernatant was concentrated and dried under vacuum at 35 °C in a vacuum CentriVap (Vacuum concentrator, Labconco, MO, USA).

The twelve type of interaction was performed twice and both were performed in triplicate (6 replicates). Three of them were redissolved in methanol and used for the determination of proanthocyanidins by size exclusion chromatography (SEC) and the phloroglucinolysis and other three were redissolved in MiliQ water to analyze of the molar masses of the soluble polysaccharides in the supernatant by SEC.

### 2.6. Analysis of Polysaccharides by SEC

Soluble polysaccharides that remained in the supernatant after interactions in model solutions and the distribution of their molecular weights were determined by high-resolution exclusion chromatography. The concentrated samples were redissolved in 250 µL of MiliQ water and a 20 µL volume of this solution was injected into a flow of 1 mL/min of LiNO_3_ (0.1 M) that passed through two columns, Shodex Ohpak KB-803 and KB-805 (0.8 cm × 30 cm, Showa Denkko, Tokyo, Japan), connected in series. A refractometer was used as detector (Waters 2414). The molar mass was determined using a calibration curve made with a special calibration kit (P-400, PM = 380,000; P-200, PM = 186,000; P-100, PM = 100,000; P-50, PM = 48,000; P-20, PM = 23,700; P-10, PM = 12,200; P-5, PM = 5800; Showa Denko K.K., Tokyo, Japan).

### 2.7. Winemaking (Small-Scale Vinifications)

Red grapes of the Monastrell variety were crushed and de-stemmed and then potassium metabisulfite was added (70 mg/kg of grape). All microvinifications were carried out in triplicate in 5 L tanks using 4 kg of grapes. The control microvinification followed a traditional red wine vinification process, while another two microvinifications were made in which the soluble polysaccharides were added at the beginning of the maceration process comprising PEC (100 mg/L) and MAN (8.3 mg/L). 

All wines were fermented at 22 °C using a commercial yeast (Viniferm CT007, Agrovin SA, Alcazar de San Juan, Spain) at a dose of 25 g/hL. The maceration lasted seven days and during this time the cap was punched down twice a day. At the end of alcoholic fermentation (AF) the wines were racked, bottled, and stored at 20 °C. The analysis of the phenolic compounds was carried out at the end of the alcoholic fermentation and after six months of aging in the bottle.

### 2.8. Spectrophotometric Analysis

The color intensity (CI) was calculated as the sum of the absorbance at 620 nm, 520 nm, and 420 nm. The total polyphenol index (TPI) was calculated by measuring the absorbance at 280 nm of a 1:100 diluted wine sample. Total and polymeric anthocyanins were determined using the method described by Boulton [23].

Total tannins concentration (MCPT) was calculated using the methylcellulose precipitation method according to Smith [24]. 

### 2.9. Analysis of Tannins by Phloroglucinolysis

The proanthocyanidin content was determined by the phloroglucinolysis reagent method described by Pastor del Rio and Kennedy [25] with some modifications [13].

The methanol extract resulting from the interactions in model solutions can be used directly to carry out the phloroglucinolysis reaction. However, the wine samples collected for the analyses must be purified previously. For this, 4 mL of wine was evaporated in a CentriVap concentrator (Labconco, Kansas City, MO, USA) then redissolved in 3 mL of water and passed through a C18-SPE column (1 g, Waters). The cartridge was washed with 20 mL of water and the compounds of interest were eluted with methanol, concentrated again, and then dissolved in 400 μL of methanol.

Initially, 50 µL of the methanolic extract of the proanthocyanidins were mixed with 50 µL of the phloroglucinolysis reagent, which was prepared by dissolving phloroglucinol (Sigma, Aldrich, MO, USA) (100 g/L) and ascorbic acid (Sigma, Aldrich, MO, USA) (20 g/L) in a solution of 2 N hydrochloric acid in methanol. The mixture was heated in a bath with water at 50 °C for 20 min. To quench the reaction, 100 µL of an aqueous sodium acetate solution (0.2 M) was added.

Tannins were estimated from their response factors relative to (+)-catechin, which was used as the quantitative standard. Using the phloroglucinolysis reagent, the total proanthocyanidin content, the apparent mDP and the percentage of each constitutive unit were determined. The sum of the subunits flavan-3-ol monomer and phloroglucionol adducts (mol) divided by the sum of flavan-3-ol monomers (mol) gives the mDP. The standards (+)-catechin, (−)-epicatechin, (−)-epicatechin gallate, and (−)-epigallocatechin were obtained from Extrasyntese (Genay, France). The conditions of the HPLC analyses were described by Busse-Valverde et al. [26].

### 2.10. Analysis of Tannins by SEC

Size exclusion chromatography (SEC) was also used to analyze tannins in the wines and the interactions in model solutions. This method was described by Kennedy and Taylor [27], with some adaptations described by Castro-López et al. [13].

### 2.11. Wine Sensory Analysis

Descriptive analysis aims to profile the color, aroma, and flavor-by-mouth of the wine. The panel was formed by 10 well-trained judges from our research group. The evaluation of the wines was carried out in two sessions that were held on different days under white lights in separate booths. The wine samples were presented in clear glass cups of 125 mL, coded, and arranged in random order according to Sancho et al. [28].

The intensity of wine color, flavor-by-mouth attributes (mouth intensity, quality, body, persistence, bitterness, astringency, dryness, and greenness), and aroma attributes (aroma intensity, aroma quality, fruit, vegetal) was scored on a scale of 0 to 10. On this scale, 0 indicates that the attribute has not been perceived by the panelist, while 10 means that the attribute is perceived very intensely.

### 2.12. Statistical Analysis

The statistical analysis of the results was made using the statistical package Statgraphics Centurion XVI. An analysis of variance (ANOVA) was first made to determine differences among samples. When there were significant differences, LSD was used to separate the means with a confidence level of 95% (*p*-value of 0.05).

## 3. Results

### 3.1. Analysis of Tannins Remaining in Solution (Measured by Phloroglucinolysis) after the Interactions with Polysaccharides in the Experiments with Model Solutions

As a first part of the experiment, commercial skin and seed tannins were first led to interact in model solution with three different polysaccharide samples and two commercial polysaccharides: PEC, MAN, and CW-PS. The material solubilized from cell walls after stirring them in a model solution for 90 min to determine whether they formed soluble complexes or whether tannins were lost through precipitation. A second part of the experiment consisted of bringing tannins into contact with cell walls in the absence or presence of the two commercial polysaccharides to determine whether they might limit tannin adsorption to cell walls. 

Table 1 shows the concentration and characteristics of the tannins remaining in solution after their interaction with the different polysaccharides and also the results of the concentration of the skin tannins remaining in solution when skin cell walls and polysaccharides were both present in the buffer solution.

Previous studies have shown that tannins and some polysaccharides form complexes that remain stable in colloidal suspension [9,15]. Moreover, some nephelometric studies have given evidence for the disrupting effect of some polysaccharides on tannins aggregation and, more importantly, on their aggregation by proteins [15].

In the present study, we worked with two different polysaccharides: a highly esterified pectin and mannan, a neutral polysaccharide. Pectins are some of the most important components in grapes cell walls and some studies have shown that they present a high affinity for tannins [9]. Mannose polymers exhibit biological activity and beneficial health characteristics, such as providing dietary fiber and satiety [29]. Linear mannans are homopolysaccharides composed of linear main chains of α-1, 6-linked D-mannose residues. The structure of mannan from *Saccharomyces cerevisiae* has been reported as presenting a backbone, composed of α-1,6-linked mannose residues, 83% branched at O-2 by single mannose residues, as well as oligosaccharide side chains mostly in the form of tri-, di-, mono-, and tetramers. Longer side chains, penta- to heptamers are present in lesser amounts. This was confirmed by Nuclear Magnetic Resonance (NMR) spectra that showed that they were formed mostly by mannose residues [30,31]. In wines, mannoproteins (proteoglycans formed of different proportions of protein and D-mannose) have been extensively used to improve several taste characteristics of red wines in which they: appear to have a protective effect on the monomeric anthocyanin content [32], soften the taste [33], improve protein stability and reduce the quantity of bentonite [34], and also promote tartrate stability [35]. However, as mentioned above, this compound is a glycoprotein and the protein part could be responsible for some unexpected results [18,19,36], which is why we chose to test only the polysaccharide fraction of *Saccharomyces cerevisiae* glycoprotein MAN was added at a lower concentration than PEC. When looking at the bibliography on Monastrell wine polysaccharides and oligosaccharides, we observed that mannose residues were always present at a lower concentration than galacturonic acid residues in the wine oligosaccharides and the content of RGII normally exceed that of mannoproteins [17,37]. Based on these previous results, we decided to work with a smaller concentration of mannan than pectin.

As regards to the two commercial polysaccharides, when they were added with tannins to the buffer solution, the tannin concentration did not change, indicating that no precipitation occurred in the reaction and that any aggregates formed between tannins and these polysaccharides are basically soluble. 

We also studied the effect of the interaction between the skin tannins and the soluble material that resulted from stirring cell walls in a model solution (CW-PS) without the presence of the insoluble CW. Vidal et al. [38] stated that some characteristics of wine soluble cell wall components are similar to those of pectin isolated from citrus, whose hydrocolloid properties improve the mouthfeel and texture of foods. According to Gao et al. [39,40], CW-PS in wine are mostly formed from polysaccharides arising from the degradation of cell wall components due to mechanical and enzymatic actions, mainly rhamnogalacturonan I (RG I), rhamnogalacturonan II (RG II), xyloglucan (XG), arabinogalactan (AG), and arabinogalactan-protein (AGP). Our results showed that this material did not behave in the same way as the commercial polysaccharides, since it caused a substantial decrease in the concentration of skin tannins in solution (46.5%) and the formation of a precipitate. Similar findings were observed by Bindon et al. [2], who noted that, in the interaction of the mesocarp or skin cell wall solubilized in the supernatant with tannins, the tannin concentration was reduced and a visible precipitate was formed. The cause of this reduction in tannin concentration and precipitate formation was mainly attributed by these authors to the formation of insoluble protein–tannin complexes and their consequent precipitation, especially in the case of mesocarp cell walls. On the other hand, they found that, in the precipitates from the skin cell walls extract, tannins were found to be mainly associated with insoluble polysaccharides although small quantities of proteins were also observed in the precipitate. The precipitated tannins were of high molecular mass, which justifies our observations concerning the reduced concentration of tannins and of the decrease in the mDP of the tannins remaining in solution.

When we simultaneously added grape skin tannin and skin cell walls to the solution, there was an 83.6% reduction in the concentration of the tannins remaining in solution. This effect was not unexpected and was already observed by Bautista-Ortín et al. [41] working with different varieties (Monastrell and Syrah) and different tannins, and by Bindon et al. [4]. The addition of soluble polysaccharides to the medium reduced the interactions between skin tannins and the cell walls, increasing the concentration of tannins in solution, pointing to competition between the cell wall bound and soluble polysaccharides for the skin tannins. As a result, losses caused by cell wall adsorption were reduced, although the adsorption to cell walls seems to be the predominant interaction. 

The mean degree of polymerization (mDP) of the skin tannin remaining in solution was reduced after the reaction with the cell walls, particularly in the experiments in which commercial soluble polysaccharides were also present. Tannins with the highest degree of polymerization are those with the highest affinity for cell walls [13,14,42,43], so those remaining in the solution presented a lower mDP. The large decrease observed when the commercial polysaccharides were present may indicate that a slight degree of precipitation of high molecular weight tannin-commercial polysaccharide complexes could have occurred. Since direct interactions with the commercial polysaccharides led to no precipitation, any precipitation, if it existed, can only be attributed to the CW-PS released during this experiment. Mateus et al. [15] stated that the masses and structures of colloidal tannin−polysaccharide complexes depend on the degree of tannin polymerization; short tannins and polysaccharides are aggregated into loose oligomeric structures whose sizes are comparable to a single polysaccharide molecule. Tannins longer than 10 nm (high mDP tannins) and polysaccharides aggregate into larger microgel-like particles, whose sizes exceed 200 nm and may sometimes precipitate.

The percentage of galloylation did not change when the grape skin tannin interacted with the commercial soluble polysaccharides or with the cell walls, but it did decrease significantly with CW-PS + SkinTan and when both cell walls and soluble polysaccharides were present in the interaction. It is possible that galloylated tannins present a high affinity for the proteins present in the soluble material since these tannins have always been considered as the cause of a high degree of astringency in wines and could explain why it decreases in the presence of solubilized proteins. 

The percentage of epigallocatechin in the tannins remaining in solution decreased significantly in all the experiments. Trihydroxylated subunits may present a higher affinity for interactions since epigallocatechin has an additional hydroxyl group on ring B that provides an extra site for hydrogen bonding to occur [44].

When seed tannins were used, differences could be observed (Table 2). The addition of soluble polysaccharides to the medium did not decrease the grape seed tannin content in solution with any of the polysaccharides, indicating the formation of soluble complexes.

When seed tannins were added to the soluble material obtained from the cell walls stirred in model solution, the seed tannins in the solution decreased, similarly to that described for skin tannins, although to a lower degree in proportion. Several studies have shown that the reactivity of tannins with different compounds increases with the molecular weight [45,46,47,48]. In the same way, Mateus et al. [15] also stated that the affinity of tannins and proteins increased with tannin mDP, which is why a greater reduction in tannins was seen when skin tannins were used. 

On the other hand, when the interaction between the grape seed tannins and the cell walls was studied, there was a 64.8% reduction of the tannins remaining in solution, a lower decrease than that observed in the case of skin tannins. This finding was similar to those of Bindon [4], who also recorded that tannins from grape skin had a greater affinity for Syrah grape cell walls than tannins from seeds. 

The addition of soluble polysaccharides to the model solution containing seed tannin and cell walls significantly reduced the adsorption of tannin by these cell walls, with no significant difference being observed between the soluble polysaccharides added, although the reduction was higher than that observed with skin tannins. 

The average degree of polymerization of the seed tannin was reduced in the presence of soluble polysaccharides and cell walls, and even more so when both were in contact with the tannin, which coincided with the results obtained with grape skin tannin.

The percentage of galloylation of the seed tannin did not change in the presence of the commercial polysaccharides but decreased in the presence of CW-PS. Those tannins remaining in the solution presented a reduced %Gal in the presence of cell walls, especially if commercial polysaccharides were also present.

### 3.2. Analysis of the Tannins Measured by SEC in Model Solutions

SEC completes the information obtained with the phloroglucinolysis method, which cannot detect those tannins that cannot be depolymerized by phloroglucinol. This technique gives information about the mass distribution of the tannins in solution and improves our knowledge as to how phenolic compounds are affected by the treatments.

**Figure 1:**
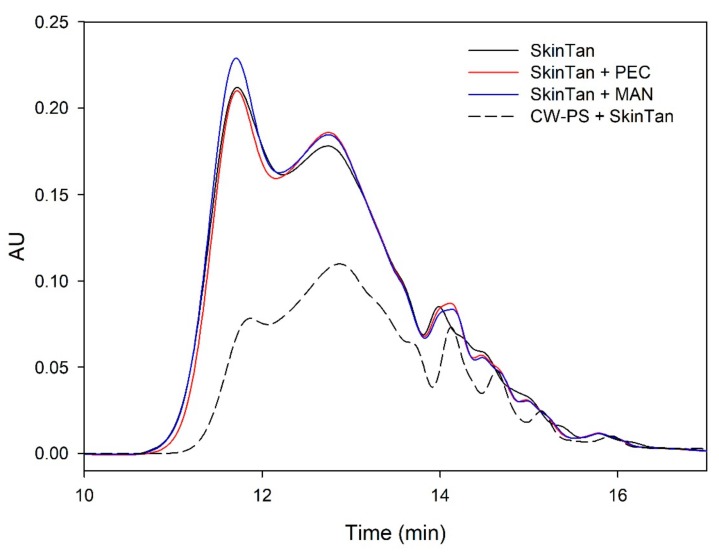
Analysis of the mass distribution of skin tannins by SEC. Comparison of the skin tannins in the absence (SkinTan) and presence of PEC, MAN, and PS-CW.

**Figure 2:**
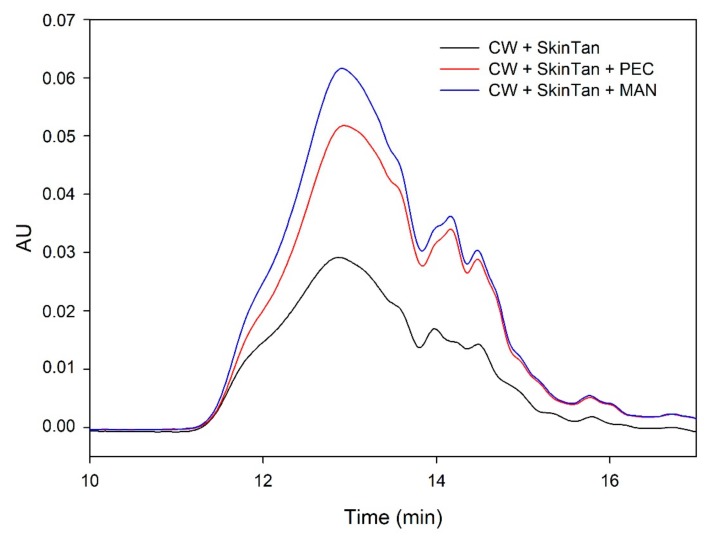
Analysis of the mass distribution of skin tannins in presence of CW by SEC. Comparison of the skin tannins in the absence (CW + SkinTan) and presence of PEC and MAN.

Figure 1 and Figure 2 and Table 3 show the mass distribution of the skin tannins that remained in the solution in the different experiments with model solutions. It can be seen that the addition of the two commercial polysaccharides to the tannin solution led to slight changes in the profile of the mass distribution of these tannins. These results confirm that polysaccharide–skin tannin complexes do not form structures that precipitate under our conditions, while the slightly lager areas presented by the tannin–polysaccharide complexes could be due to an increase in the overall particle size of the complexes, as observed by Li et al. [29]. This phenomenon was not observed in the mDP values, possibly due to that tannin–polysaccharide complexes could be formed mainly with oxidized tannins, which do not show a response by the phloroglucinolysis method.

The results of the interaction between the skin tannin and the soluble material released from cell walls (CW-PS + SkinTan) confirmed that the soluble material dramatically decreases the content of tannins in solution, which agrees with our phloroglucinolysis analysis results.

Figure 2 shows the profile of the tannins remaining in solution after the interactions of skin tannins and cell walls, with or without the commercial soluble polysaccharides. As already observed by the phloroglucinolysis technique, the presence of the soluble polysaccharides increased the concentration of skin tannins in solution, probably as a result of competition with cell walls. In accordance with the results obtained in phloroglucinolysis, MAN was the soluble polysaccharide that maintained the highest amount of skin tannin in solution after the interaction, closely followed by the amounts found when PEC was used. It was surprising that MAN and PEC performed similarly, given the different concentrations used for each of them. Regarding the possible causes behind PEC and MAN resulting in similar capacities of interaction, it can be hypothesized that the low pH of the model solution led to demethylation of the highly methylated pectin [49], which, in turn, lowers its affinity for tannins [7].

**Figure 3:**
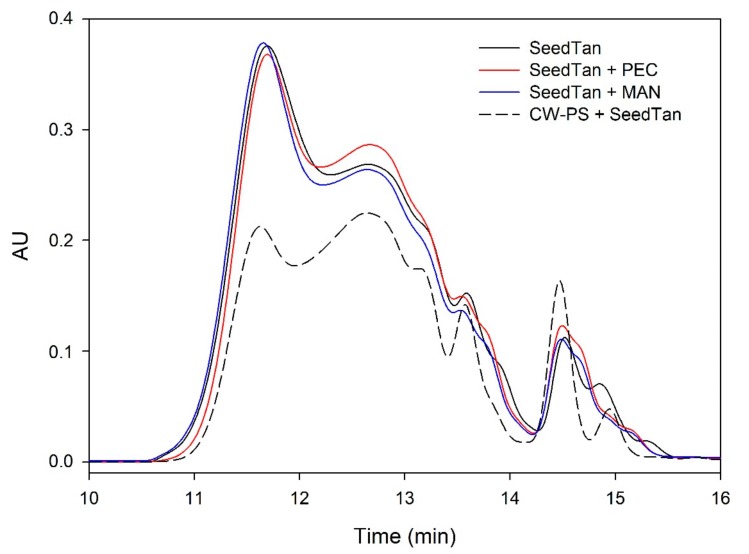
Analysis of the mass distribution of seed tannins by SEC. Comparison of the seed tannins in the absence (SeedTan) and presence of PEC, MAN, and PS-CW.

**Figure 4:**
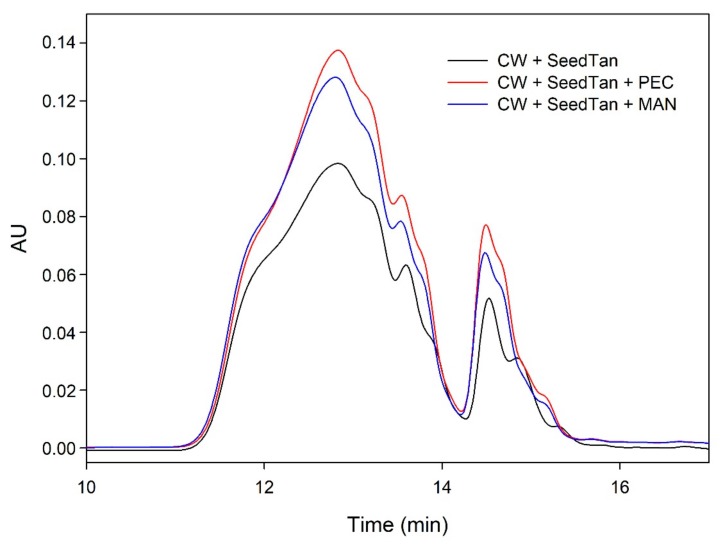
SEC analysis of the mass distribution of seed tannins in the presence of CW. Comparison of the seed tannins in the absence (CW + SeedTan) and presence of PEC and MAN.

Figure 3 and Figure 4 and Table 4 show the mass distribution of the seed tannins that remained in solution in the different experiments with the model solutions. Figure 3 shows that the addition of soluble polysaccharides to seed tannins hardly changed the profile of the mass distribution of the seed tannin in solution. As with the grape skin tannin, in the presence of CW-PS, a pronounced decrease in the tannins in solution was observed (especially of the polymeric and oligomeric compounds), confirming the results found with the phloroglucinolysis analysis. 

### 3.3. Analysis of the Soluble Polysaccharides Released in the Tests with Model Solutions by SEC

**Figure 5:**
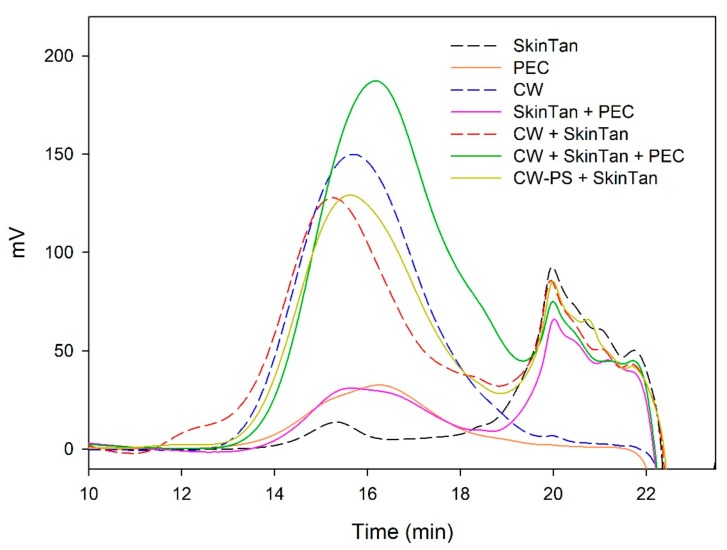
Analysis of the mass distribution of polysaccharides by SEC. Comparison of the polysaccharides released in the interaction tests between SkinTan, PEC, CW, and CW-PS.

**Figure 6:**
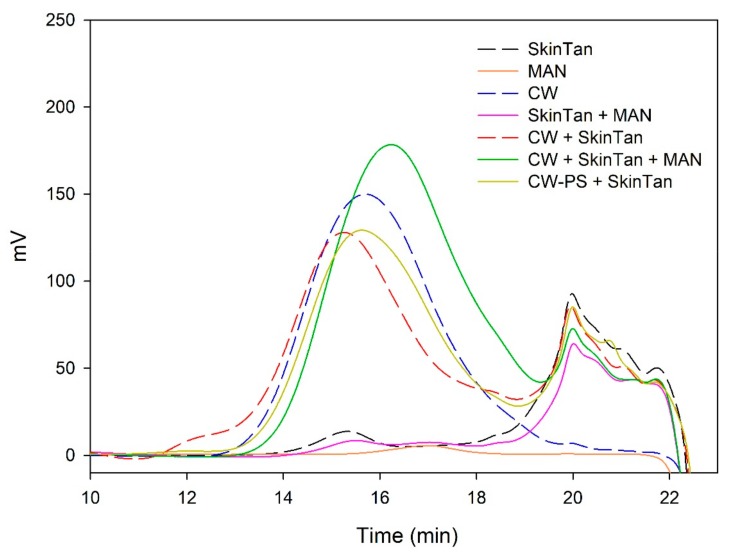
Analysis of the mass distribution of polysaccharides by SEC. Comparison of the polysaccharides released in the interaction tests between SkinTan, MAN, CW, and CW-PS.

**Figure 7:**
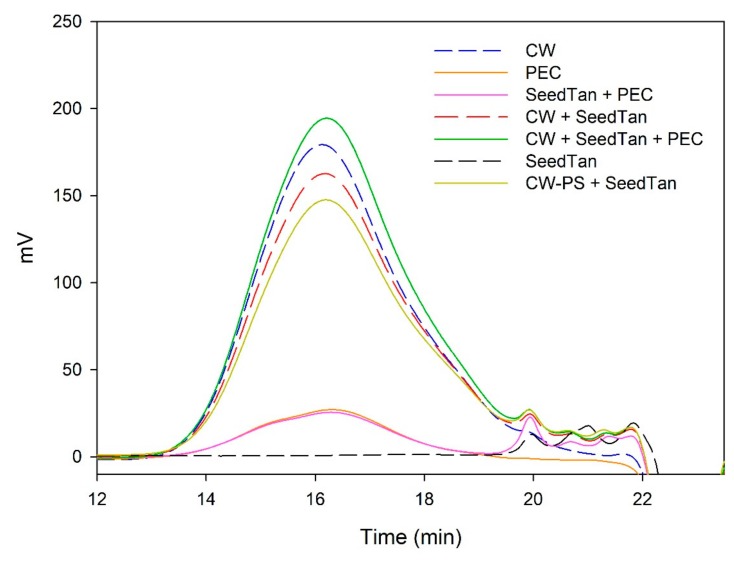
Analysis of the mass distribution of polysaccharides by SEC. Comparison of the polysaccharides released in the interaction tests between SeedTan, PEC, CW, and CW-PS.

**Figure 8:**
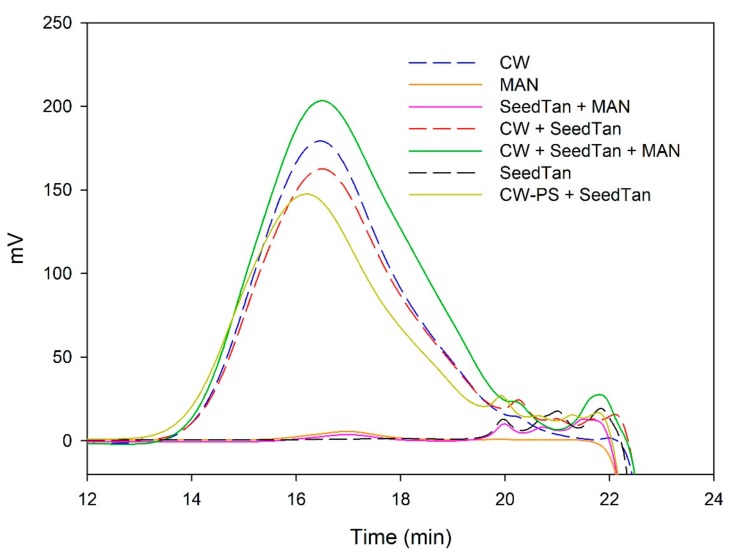
Analysis of the mass distribution of polysaccharides by SEC. Comparison of the polysaccharides released in the interaction tests between SeedTan, MAN, CW, and CW-PS.

To further analyze the role played by the polysaccharides solubilized from cell walls in the precipitation of skin and seed tannins, we have studied the changes observed in their concentration after the different interactions using SEC. Figure 5, Figure 6, Figure 7 and Figure 8 and Table 5 show the profile of the soluble polysaccharides in the model solution after the different interactions. In this study, the area corresponding to the commercial polysaccharides (PEC and MAN) and the area resulting from the interaction between commercial polysaccharides and tannins (PEC + SkinTan, PEC + SeedTan, MAN + SkinTan, and MAN + SeedTan) clearly showed that, similarly to that observed when tannins were analyzed by SEC, the concentration of polysaccharides did not change, confirming the absence of polysaccharide precipitation and that the complexes formed between these commercial polysaccharides and tannins are soluble. 

We also studied the concentration of the polysaccharides remaining in solution after the reaction of suspended CWs and the tannins in the presence or absence of the commercial PEC and MAN. The area of these polysaccharides (CW) in the absence of tannins was very large compared with the area representing the added commercial polysaccharides. When the skin or seed tannins were added to the model solution containing the suspended cell walls and were allowed to interact for 90 min, the area slightly decreased in the case of skin tannins (10%) and in the case of seed tannins (7%), but more so when only the CW-PS (the solution after stirring the cell walls followed by the elimination of the insoluble and suspended cell walls) interacted with the tannins, especially when seed tannins were used (15%). We hypothesized that in the presence of suspended cell walls, part of the tannins binds to this material (this was already observed to be the predominant reaction) and, therefore, does not interact with the soluble polysaccharides. Only when the soluble polysaccharides are present do all the added tannins interact with them and the probability of precipitation of this soluble material increases. Bindon et al. [2] stated that although pectic polysaccharides have the capacity to interact with tannins in solution to form a stable, soluble complex, coprecipitation of soluble material and tannins has also been observed probably resulting from complexes formed with the polysaccharides. Riou et al. [50] reported that with Rhamnogalacturonan (II) dimers a co-aggregation with tannins was observed and that could lead to precipitation. 

However, it is important to note that the decrease in the concentration of polysaccharides after the interaction with tannins is very small compared with the decrease observed in the concentration of tannins (Figure 1, Figure 2, Figure 3 and Figure 4). This difference can only be attributed to the fact that other compounds beside polysaccharides are responsible for a large part of tannin precipitation (probably proteins, which although present in low concentrations may play an important role). 

When the commercial polysaccharides PEC and MAN were added to the solution containing the suspended cell walls and the tannins, the area corresponding to the polysaccharides increased significantly, indicating that these commercial products did not precipitate in the medium, in agreement with previous results. 

### 3.4. The Role of Soluble Polysaccharides in the Chromatic and Sensory Characteristics of Finished Wines

After the studies made with model solutions, it was decided to gain more knowledge of the behavior of these soluble polysaccharides in real vinifications, to ascertain if using them just after crushing (instead of using them in finished wines, as it is common with other polysaccharides such as mannoprotein) could reduce the loss of tannins and improve the chromatic and sensory characteristics of a wine. 

We already knew that the transfer rate of phenolic compounds from grape to must is limited due to, among other reasons, the interactions that occur between phenolic compounds and the skin and pulp cell walls present in the must in large concentrations, interactions that prevent these pigments from contributing to the final wine phenolic content [2,3]. Another potential mechanism has been identified in which extracted grape tannins may be lost from must/wine during vinification, and this is as a precipitate with solubilized grape proteins [51,52]. These authors stated that although protein is, in general, a minor component in terms of total concentration, losses of tannins via precipitation with proteins could be in the order of 50% of available tannins.

If the capacity of polysaccharides to interact with tannins in solution to form a stable soluble complex can be confirmed in wines, this could be used to reduce polyphenol fining during vinification (by limiting must protein–polyphenol precipitation and/or competing with the adsorption onto cell walls). These interactions could also modulate wine astringency since the formation of tannin and polysaccharide complexes may influence their reactivity with salivary proteins and lead to changes in the perception of astringency [53]. As an example, soluble pectins have been described as reducing the astringency of persimmon [54] and other carbohydrates such as xanthan or Arabic gum could also have the same effect [55].

Therefore, we added the two studied polysaccharides at the beginning of a red wine vinification to favor competition between the adsorption of tannins by the insoluble cell wall material in suspension in the must and their reaction with proteins. For this, wine chromatic characteristics were measured at the end of alcoholic fermentation and after six months in the bottle (Table 6).

With regards to the chromatic parameters, the color intensity at the end of alcoholic fermentation and after six months in the bottle of the two wines to which soluble polysaccharides were added just after crushing significantly increased. The wine with added PEC showed the highest color intensity.

The total polyphenol index was significantly higher in the wines with added polysaccharides than in the control wine, both at the end of alcoholic fermentation and after six months in the bottle, with no significant differences between the polysaccharides. 

The addition of soluble polysaccharides significantly increased total anthocyanins in the wines. At the end of the alcoholic fermentation, there were significant differences among all the wines, the wines with added PEC obtaining the highest results after six months in the bottle, total anthocyanins decreased slightly due to polymerizations, but the differences with the control wine were maintained and no differences between the PEC-containing and the MAN-containing wines were observed. 

In terms of the MCPT, it was observed that the addition of soluble polysaccharides led to wines with significantly higher tannin values than control wines, almost doubling the concentration. After six months in the bottle, the vinification with added mannan continued to show a significantly higher tannin concentration than the control wine, although the tannin concentration of this wine decreased much more than in the control vinification or wine with added PEC. It could be hypothesized that this fall in the total tannin values could be due to the precipitation of high molecular tannin–polysaccharide aggregates.

The concentrations of total tannins measured by the phloroglucinolysis method are shown in Table 7. In the analysis carried out in the wines at the end of AF, the results clearly showed that the addition of both soluble polysaccharides led to a significant increase in the tannin concentration, the highest values being found in the pectin-added wine, with more than double the total tannins measured in the control wine, and mannan-added wines also showed a higher concentration of tannins than control wines. Similarly to the results for MCPT, the mannan-added wines showed a notable loss of tannins after six months in the bottle. This may be due to the complexes that these tannins form with these soluble polysaccharides and which precipitate over time. 

The mPD of tannins increased with the addition of soluble polysaccharides in both wines, at the end of AF and after six months in the bottle. This finding was to be expected since the most polymerized tannins are the most susceptible to binding to cell walls and precipitating with proteins. When the soluble polysaccharides were added, they competed with the cell walls and proteins to bind to these tannins so that the amount of polymeric tannins (and hence mDP) in the resulting wine increased.

**Figure 9:**
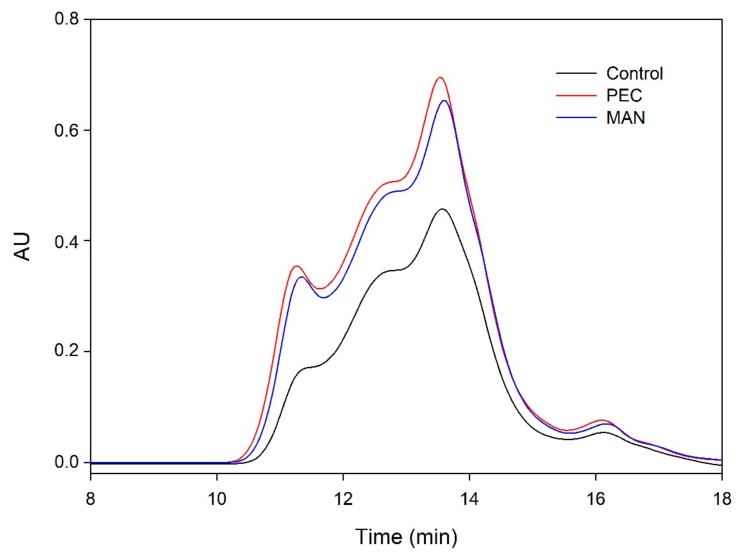
Mass distribution of the proanthocyanidins of the final wines analyzed by SEC at the end of alcoholic fermentation.

**Figure 10:**
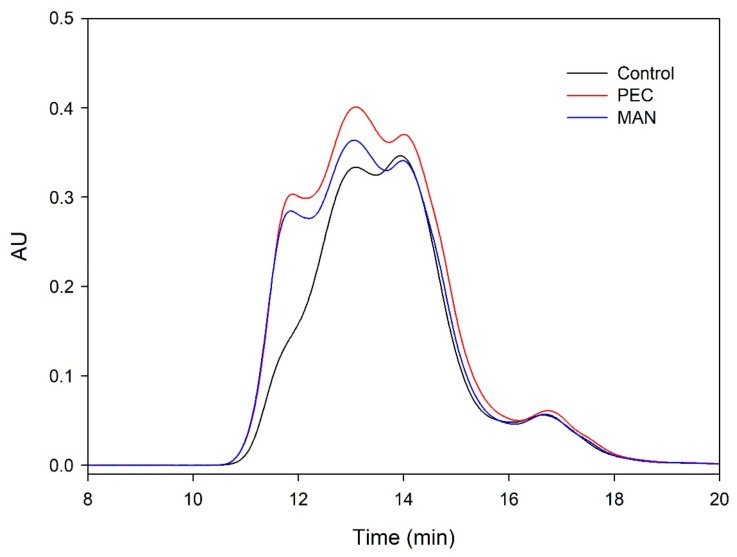
Mass distribution of the proanthocyanidins of the final wines analyzed by SEC after six months of aging in the bottle.

The distribution of the molecular weights of the proanthocyanidins from the different microvinifications obtained in the SEC analysis is shown in Figure 9 and Figure 10.

When the analysis was carried out at the end of AF (Figure 9), the area of the wines supplemented with polysaccharides was higher for all the molecular masses. The level of polymeric tannins was one of the fractions that was clearly higher in the presence of polysaccharides, coinciding with the higher mDP found in these wines. This profile changed during wine aging (Figure 10), when polymerization reactions developed, changing the molecular weight distribution profile. After six months of aging in the bottle, the differences between vinifications decreased.

**Figure 11:**
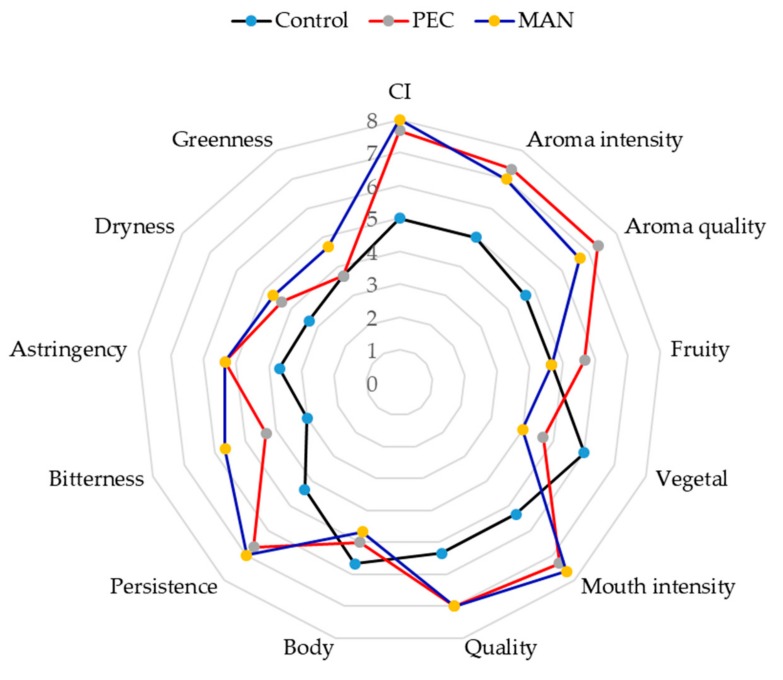
Results of the descriptive analysis performed by ten panelists for wines after six months in the bottle.

Then, a sensory analysis was conducted with the wines that had been six months in the bottle (Figure 11). The visual properties of the wines made with added PEC and MAN scored higher than the control wines, which coincides with the results obtained in the colorimetric analysis.

As for the aroma descriptors, the intensity and quality of the aroma of the polysaccharides-supplemented wines scored higher than the control wine, the highest values being observed in the PEC wines, followed by MAN wines. Fruit aroma was also better scored in wines with soluble polysaccharides, whereas the score for vegetal aroma was higher in the control wine. Mitropoulou et al. [56] studied the effect of commercial tannin extracts, a natural wine polysaccharide extract, pectin, and arabinogalactan on the headspace release of selected aroma compounds from a “model wine” solution observing that, in general, the volatility of esters was generally increased upon tannin addition except at very high addition levels (10 g/L). Both arabinogalactan and pectin addition at low concentrations increased the volatility of the studied aroma compounds, while at higher concentrations pectin exhibited a different behavior by salting out hydrophobic compounds in the vapor phase. 

Moreover, Saenz-Navajas et al. [57] stated that sensory quality was primarily related to wines without defective aroma and secondarily to the presence of some nonvolatile components, among them, proanthocyanidins linked to polysaccharide.

Regarding in-mouth parameters, wines with added polysaccharides scored higher for astringency and bitterness, probably due to the higher tannin content, although persistence, intensity, and mouthfeel quality had higher scores in the wines containing the polysaccharides. It has been described that polysaccharides in wine significantly increase the ‘fullness’ sensation and can potentially add ‘mellowness’ to wines [16] and this could compensate the higher astringency sensation caused by the higher tannin concentration.

## 4. Conclusions

The results confirmed that skin and seed tannins interact strongly with both the insoluble and soluble cell wall material present in model solutions, the concentration of tannins being substantially lower after the interaction. This loss would be due to the binding of tannins to the suspended cell wall material and to the formation of insoluble complexes with some components of the soluble cell wall material. The results also showed that polysaccharides do not seem to largely contribute to the composition of these precipitates since their concentration in solution did not decrease as much as the concentration of tannins did as a result of the interactions. Other compounds (probably proteins) might be responsible for the precipitation of the tannins. The addition of two commercial polysaccharides led to the formation of soluble complexes, reducing the tannin losses. This effect was also observed when the polysaccharides were used in real vinifications. The addition of the commercial polysaccharides at the beginning of winemaking led to wines with higher phenolic content and improved chromatic characteristics, which remained stable after six months in the bottle, as seen from the highest scores reached in the sensory analysis and, especially when pectin was used. 

## Figures and Tables

**Table 1:** Effect of the addition of soluble polysaccharides on the interaction between skin tannin and cell walls. Characterization and quantification of tannins in solution.

Samples	TT mg/L	mPD	%Gal	% Epigal
SkinTan	708.31 ± 42.89b ^1^	3.76 ± 0.01d	2.34 ± 0.01b	9.15 ± 0.20c
SkinTan + PEC	701.32 ± 17.85b	3.63 ± 0.00b	2.31 ± 0.01b	8.37 ± 0.09b
SkinTan + MAN	715.94 ± 19.87b	3.71 ± 0.02c	2.31 ± 0.02b	8.50 ± 0.10b
SkinTan + CW-PS	378.79 ± 19.61a	3.28 ± 0.01a	2.03 ± 0.18a	8.01 ± 0.12a
CW + SkinTan	116.10 ± 4.99a	3.08 ± 0.05c	2.34 ± 0.06b	8.85 ± 0.34c
CW + SkinTan + PEC	190.73 ± 12.19b	2.72 ± 0.01a	2.18 ± 0.02a	7.04 ± 0.13a
CW + SkinTan + MAN	202.40 ± 6.65b	2.91 ± 0.07b	2.15 ± 0.03a	8.17 ± 0.24b

Abbreviations: SkinTan: tannin from grape skin; CW: cell wall; PEC: esterified pectin; M: mannan; TT: total tannins; DPm: mean degree of polymerization; %Gal: % of galloylation; %Epigal: % of epigallocatechin. ^1^ Different letters in the same column and for each type of interaction test mean statistically significant differences (*p* < 0.05).

**Table 2:** Effect of the addition of soluble polysaccharides on the interaction between seed tannins and cell walls. Characterization and quantification of tannins in solution.

Samples	TT mg/L	mPD	%Gal
SeedTan	1426.72 ± 102.62b ^1^	2.82 ± 0.01d	14.61 ± 0.01b
SeedTan + PEC	1469.70 ± 142.31b	2.71 ± 0.01b	14.27 ± 0.03b
SeedTan + MAN	1533.75 + 143.72b	2.77 ± 0.04c	14.30 ± 0.45b
SeedTan + CW-PS	1071.31 ± 40.14a	2.50 ± 0.02a	13.21 ± 0.07a
CW + SeedTan	502.40 ± 26.73a	2.39 ± 0.06b	13.17 ± 0.25b
CW + SeedTan + PEC	636.22 ± 63.79b	2.20 ± 0.04a	11.70 ± 0.28a
CW + SeedTan + MAN	662.84 ± 12.92b	2.29 ± 0.04a	12.12 ± 0.20a

Abbreviations: SeedTan: tannin from grape seed; CW: cell wall; PEC: esterified pectin; M: mannan; TT: total tannins; DPm: mean degree of polymerization; %Gal: % of galloylation. ^1^ Different letters in the same column and for each type of interaction test mean statistically significant differences (*p* < 0.05).

**Table 3:** Total area measured in the SEC analysis for the skin tannin in the model solution and area corresponding to high molecular mass (F1), medium molecular mass (F2), and those corresponding to low molecular mass phenolic compounds (F3).

Samples	Total Area	F1	F2	F3
SkinTan	0.4914	0.1545 (31.5) ^1^	0.2371 (48.3) ^1^	0.0957 (19.5) ^1^
SkinTan + PEC	0.4830	0.1441 (29.8)	0.2405 (49.8)	0.0944 (19.6)
SkinTan + MAN	0.4989	0.1611 (32.3)	0.2403 (48.2)	0.0934 (18.7)
CW-PS + SkinTan	0.2609	0.0439 (16.8)	0.1422 (54.5)	0.0725 (27.8)
CW + SkinTan	0.0531	0.0038 (7.2)	0.0363 (68.4)	0.0125 (23.6)
CW + SkinTan + PEC	0.1165	0.0085 (7.3)	0.0665 (57.1)	0.0406 (34.9)
CW + SkinTan + MAN	0.1332	0.0106 (8.0)	0.0783 (58.8)	0.0433 (32.5)

^1^ The number in parentheses indicates the percentage of the total area. F1: compounds with a molecular mass ranging between 840,000 and 30,000 g/mol, corresponding to compounds eluting from 10 to 11.8 min in the SEC analysis. F2: compounds with a molecular mass ranging between 30,000 and 1000 g/mol, corresponding to compounds eluting from 11.8 to 13.6 min in the SEC analysis. F3: compounds with a molecular mass lower than 1000 g/mol and corresponding to compounds eluting from 13.6 and 16 min in the SEC analysis.

**Table 4:** Total area measured in the SEC analysis for the seed tannin in the model solution and area corresponding to high molecular mass (F1), medium molecular mass (F2), and low molecular mass phenolic compounds (F3).

Samples	Total Area	F1	F2	F3
SeedTan	0.6813	0.2857 (41.9) ^1^	0.3262 (47.9) ^1^	0.0625 (9.2) ^1^
SeedTan + PEC	0.6711	0.2647 (39.5)	0.3395 (50.6)	0.0599 (8.9)
SeedTan + MAN	0.6624	0.2895 (43.7)	0.3123 (47.2)	0.054 (8.2)
CW-PS + SeedTan	0.4585	0.1303 (28.4)	0.2676 (58.4)	0.0559 (12.2)
CW + SeedTan	0.1763	0.0321 (18.2)	0.1163 (66.0)	0.0258 (14.6)
CW + SeedTan + PEC	0.2348	0.0398 (17.0)	0.1582 (67.4)	0.0339 (14.4)
CW + SeedTan + MAN	0.2232	0.0423 (19.0)	0.1483 (66.5)	0.0298 (13.4)

^1^ The number in parentheses indicates the percentage of the total area. F1: compounds with a molecular mass ranging between 840,000 and 30,000 g/mol, corresponding to compounds eluting from 10 to 11.8 min in the SEC analysis. F2: compounds with a molecular mass ranging between 30,000 and 1000 g/mol, corresponding to compounds eluting from 11.8 to 13.6 min in the SEC analysis. F3: compounds with a molecular mass lower than 1000 g/mol and corresponding to compounds eluting from 13.6 and 16 min in the SEC analysis.

**Table 5:** Total area measured in the SEC analysis for the soluble polysaccharides in the model solution after the interaction tests.

Samples	SkinTan	SeedTan
CW	513.4	513.4
Tan	40.0	7.1
PEC	87.9	87.9
MAN	12.2	12.2
PEC + Tan	94.9	74.8
MAN + Tan	27.1	9.2
CW-PS + Tan	414.2	478.8
CW + Tan	419.6	520.2
CW + Tan + PEC	595.9	614.9
CW + Tan + MAN	563.3	672.0

**Table 6:** Results of the chromatic analysis of wines treated with soluble polysaccharides at the end of alcoholic fermentation and after six months of aging in the bottle.

End of AF	CI	TPI	TA (mg/L)	PolA (mg/L)	MCPT (mg/L)
Control	5.1 ± 0.06a ^1^	20.7 ± 0.15a	274.5 ± 17.95a	8.5 ± 0.15a	351.9 ± 16.40a
PEC	7.4 ± 0.15c	32.2 ± 1.30b	378.2 ± 15.55c	14.5 ± 1.30b	726.3 ± 21.94b
MAN	7.2 ± 0.15b	31.8 ± 0.95b	347.8 ± 10.00b	15.3 ± 0.95b	733.0 ± 47.37b
**6 months**					
Control	4.4 ± 0.22a	22.0 ± 0.63a	225.5 ± 10.10a	10.9 ± 1.23a	339.5 ± 30.80a
PEC	7.2 ± 0.26c	32.0 ± 0.58b	299.3 ± 13.66b	22.1 ± 2.57b	439.8 ± 6.12b
MAN	6.5 ± 0.21b	30.9 ± 0.84b	307.0 ± 19.06b	19.9 ± 0.57b	388.7 ± 33.78ab

CI: color intensity; TA: total anthocyanins; TPI: total polyphenol index; PolA: polymeric anthocyanins; MCPT: total tannins measured by methylcellulose precipitation method. ^1^ Different letters in the same column and for each studied time mean statistically significant differences (*p* < 0.05).

**Table 7:** Concentration and composition of the tannins of wines treated with commercial soluble polysaccharides analyzed by the phloroglucinolysis method.

End of AF	TT Phloro (mg/L)	mPD	%Gal	% Epigal
Control	223.9 ± 16.05a ^1^	3.5 ± 0.21a	2.90 ± 0.01b	13.53 ± 0.13a
PEC	516.5 ± 5.78c	4.5 ± 0.19b	2.83 ± 0.04a	15.01 ± 0.21b
MAN	459.1 ± 1.02b	4.4 ± 0.01b	2.93 ± 0.01b	18.08 ± 0.45c
**6 months**				
Control	183.0 ± 10.01a	3.6 ± 0.29a	2.90 ± 0.60a	13.69 ± 2.22a
PEC	374.7 ± 57.04c	4.1 ± 0.09b	2.33 ± 0.18a	15.33 ± 0.95a
MAN	266.3 ± 24.16b	4.2 ± 0.12b	3.06 ± 0.29a	14.60 ± 0.45a

TT phloro: total tannins measured by phloroglucinolysis; mDP: mean degree of polymerization; %Gal: % of galloylation; % Epigal: % of Epigallocatechin. ^1^ Different letters in the same column and for each studied time mean statistically significant differences (*p* < 0.05).

## References

[1] Hazak J.C., Harbertson J.F., Lin C.H., Ro B.H., Adams D.O. (2004). The phenolic components of grape berries in relation to wine composition. Acta Hortic..

[2] Bindon K.A., Li S., Kassara S., Smith P.A. (2016). Retention of proanthocyanidin in wine-like solution is conferred by a dynamic interaction between soluble and insoluble grape cell wall components. J. Agric. Food Chem..

[3] Osete-Alcaraz A., Bautista-Ortín A.B., Ortega-Regules A., Gómez-Plaza E. (2019). Elimination of suspended cell wall material in musts improves the phenolic content and color of red wines. Am. J. Enol. Vitic..

[4] Bindon K.A., Smith P.A., Kennedy J.A. (2010). Interaction between grape-derived proanthocyanidins and cell wall material. 1. Effect on proanthocyanidin composition and molecular mass. J. Agric. Food Chem..

[5] Renard C.M., Baron A., Guyot S., Drilleau J.F. (2001). Interactions between apple cell walls and native apple polyphenols: Quantification and some consequences. Int. J. Biol. Macromol..

[6] Lin Z., Fischer J., Wicker L. (2016). Intermolecular binding of blueberry pectin-rich fractions and anthocyanin. Food Chem..

[7] Le Bourvellec C., Bouchet B., Renard C.M. (2005). Non-covalent interaction between procyanidins and apple cell wall material. Part III: Study on model polysaccharides. Biochim. Biophys. Acta.

[8] McMannus J.P., Davis K.G., Beart J.E., Gaffney S.H., Lilley S.H., Haslam E. (1985). Polyphenol interactions. I. Introduction: Some observations on the reversible complexation of polyphenols with proteins and polysaccharides. J. Chem. Soc. Perkin Trans. 2.

[9] Ruiz-García Y., Smith P.A., Bindon K.A. (2014). Selective extraction of polysaccharide affects the adsorption of proanthocyanidin by grape cell walls. Carbohydr. Polym..

[10] Zietsman A.J., Moore J.P., Fangel J.U., Willats W.G., Trygg J., Vivier M.A. (2015). Following the compositional changes of fresh grape skin cell walls during the fermentation process in the presence and absence of maceration enzymes. J. Agric. Food Chem..

[11] Zietsman A.J., Moore J.P., Fangel J.U., Willats W.G., Vivier M.A. (2015). Profiling the hydrolysis of isolated grape berry skin cell walls by purified enzymes. J. Agric. Food Chem..

[12] Bautista-Ortín A.B., Jiménez-Pascual E., Busse-Valverde N., López-Roca J.M., Ros-García J.M., Gómez-Plaza E. (2013). Effect of wine maceration enzymes on the extraction of grape seed proanthocyanidins. Food Bioprocess Technol..

[13] Castro-López L., Gómez-Plaza E., Ortega-Regules A., Lozada D., Bautista-Ortín A.B. (2016). Role of cell wall deconstructing enzymes in the proanthocyanidin–cell wall adsorption–desorption phenomena. Food Chem..

[14] Bautista-Ortín A., Ben Abdallah R., Castro-López L., Jiménez-Martínez M., Gómez-Plaza E. (2016). Technological implications of modifying the extent of cell wall-proanthocyanidin interactions using enzymes. Int. J. Mol. Sci..

[15] Mateus N., Carvalho E., Luís C., de Freitas V. (2004). Influence of the tannin structure on the disruption effect of carbohydrates on protein–tannin aggregates. Anal. Chim. Acta.

[16] Chong H.H., Cleary M.T., Dokoozlian N., Ford C.M., Fincher G.B. (2019). Soluble Cell Wall Carbohydrates and Their Relationship with Sensory Attributes in Cabernet Sauvignon Wine. Food Chem..

[17] Apolinar-Valiente R., Williams P., Romero-Cascales I., Gómez-Plaza E., López-Roca J.M., Ros-García J.M., Doco T. (2013). Polysaccharide composition of Monastrell red wines from four different Spanish terroirs: Effect of wine-making techniques. J. Agric. Food Chem..

[18] Guadalupe Z., Ayestarán B. (2008). Effect of commercial mannoprotein addition on polysaccharide, polyphenolic, and color composition in red wines. J. Agric. Food Chem..

[19] Guadalupe Z., Martínez L., Ayestarán B. (2010). Yeast mannoproteins in red winemaking: Effect on polysaccharide, polyphenolic, and color composition. Am. J. Enol. Vitic..

[20] De Vries J.A., Voragen A.G.J., Rombouts F.M., Pilnik W. (1981). Extraction and purification of pectins from alcohol insoluble solids from ripe and unripe apples. Carbohydr. Polym..

[21] DuBois M., Gilles K.A., Hamilton J.K., Rebers P.T., Smith F. (1956). Colorimetric method for determination of sugars and related substances. Anal. Chem..

[22] Osete-Alcaraz A., Gómez-Plaza E., Martínez-Pérez M.P., Weiller F., Schückel J., Willats W., Moore J., Ros-García J.M., Bautista-Ortín A.B. (2020). The impact of carbohydrate-active enzymes on mediating cell wall polysaccharide-tannin interactions in a wine-like matrix. Food Res. Int..

[23] Boulton R. (2001). The copigmentation of anthocyanins and its role in the color of red wine: A critical review. Am. J. Enol. Vitic..

[24] Smith P.A. (2005). Precipitation of tannin with methyl cellulose allows tannin quantification in grape and wine samples. Tech. Rev. AWRI.

[25] Pastor del Rio J.L., Kennedy J.A. (2006). Development of proanthocyanidins in Vitis vinifera L. cv. Pinot noir grapes and extraction into wine. Am. J. Enol. Vitic..

[26] Busse-Valverde N., Gomez-Plaza E., Lopez-Roca J.M., Gil-Muñoz R., Fernández-Fernández J.I., Bautista-Ortín A.B. (2010). Effect of different enological practices on skin and seed proanthocyanidins in three varietal wines. J. Agric. Food Chem..

[27] Kennedy J.A., Taylor A.W. (2003). Analysis of proanthocyanidins by high-performance gel permeation chromatography. J. Chromatogr. A.

[28] Sancho J., Bota E., De Castro J. (1999). Introducción al Análisis Sensorial de los Alimentos.

[29] Li S., Wilkinson K.L., Mierczynska-Vasilev A., Bindon K.A. (2019). Applying nanoparticle tracking analysis to characterize the polydispersity of aggregates resulting from tannin–polysaccharide interactions in wine-like media. Molecules.

[30] Nakajima T., Ballou C.E. (1974). Structure of the linkage region between the polysaccharide and protein parts of Saccharomyces cerevisiae mannan. J. Biol. Chem..

[31] Vinogradov E., Petersen B., Bock K. (1998). Structural analysis of the intact polysaccharide mannan from Saccharomyces cerevisiae yeast using 1H and 13C NMR spectroscopy at 750 MHz. Carbohydr. Res..

[32] Palomero F., Morata A., Benito S., Calderón F., Suárez-Lepe J.A. (2007). New genera of yeasts for over-lees aging of red wine. Food Chem..

[33] Vidal S., Francis L., Noble A., Kwiatkowski M., Cheynier V., Waters E. (2004). Taste and mouth-feel properties of different types of tannin-like polyphenolic compounds and anthocyanins in wine. Anal. Chim. Acta.

[34] Gonzalez-Ramos D., Quiros M., Gonzalez R. (2009). Three different targets for the genetic modification of wine yeast strains resulting in improved effectiveness of bentonite fining. J. Agric. Food Chem..

[35] Moine-Ledoux V., Perrin A., Paladin I., Dubourdieu D. (1997). First result of tartaric stabilization by adding mannoproteins (Mannostab™). OENO One.

[36] Del Barrio-Galán R., Pérez-Magariño S., Ortega-Heras M., Guadalupe Z., Ayestarán B. (2012). Polysaccharide characterization of commercial dry yeast preparations and their effect on white and red wine composition. LWT-Food Sci. Technol..

[37] Apolinar-Valiente R., Williams P., Mazerolles G., Romero-Cascales I., Gómez-Plaza E., López-Roca J.M., Ros-García J.M., Doco T. (2014). Effect of enzyme additions on the oligosaccharide composition of Monastrell red wines from four different wine-growing origins in Spain. Food Chem..

[38] Vidal S., Williams P., Doco T., Moutounet M., Pellerin P. (2003). The polysaccharides of red wine: Total fractionation and characterization. Carbohydr. Polym..

[39] Gao Y., Fangel J.U., Willats W.G., Vivier M.A., Moore J.P. (2015). Dissecting the polysaccharide-rich grape cell wall changes during winemaking using combined high-throughput and fractionation methods. Carbohydr. Polym..

[40] Gao Y., Fangel J.U., Willats W.G., Vivier M.A., Moore J.P. (2016). Dissecting the polysaccharide-rich grape cell wall matrix using recombinant pectinases during winemaking. Carbohydr. Polym..

[41] Bautista-Ortín A.B., Molero N., Marín F., Ruiz-García Y., Gómez-Plaza E. (2015). Reactivity of pure and commercial grape skin tannins with cell wall material. Eur. Food Res. Technol..

[42] Bautista-Ortín A.B., Martínez-Hernández A., Ruiz-García Y., Gil-Muñoz R., Gómez-Plaza E. (2016). Anthocyanins influence tannin–cell wall interactions. Food Chem..

[43] Hanlin R.L., Hrmova M., Harbertson J.F., Downey M.O. (2010). Condensed tannin and grape cell wall interactions and their impact on tannin extractability into wine. Aust. J. Grape Wine Res..

[44] Le Bourvellec C., Guyot S., Renard C.M.G.C. (2004). Non-covalent interaction between procyanidins and apple cell wall material: Part I. Effect of some environmental parameters. Biochim. Biophys. Acta.

[45] Vidal S., Williams P., O’Neill M.A., Pellerin P. (2001). Polysaccharides from grape berry cell walls. Part I: Tissue distribution and structural characterization of the pectic polysaccharides. Carbohyd. Polym..

[46] Poncet-Legrand C., Cartalade D., Putaux J.L., Cheynier V., Vernhet A. (2003). Flavan-3-ol aggregation in model ethanolic solutions: Incidence of polyphenol structure, concentration, ethanol content, and ionic strength. Langmuir.

[47] Cheynier V., Dueñas-Paton M., Salas E., Maury C., Souquet J.M., Sarni-Manchado P., Fulcrand H. (2006). Structure and properties of wine pigments and tannins. Am. J. Enol. Vitic..

[48] Dangles O., Dufour C., Andersen O.M., Markham K.R. (2006). Flavonoid-protein interactions. Flavonoids—Chemistry, Biochemistry and Applications.

[49] Diaz J.V., Anthon G.E., Barrett D.M. (2007). Nonenzymatic degradation of citrus pectin and pectate during prolonged heating: Effects of pH, temperature, and degree of methyl esterification. J. Agric. Food Chem..

[50] Riou V., Vernhet A., Doco T., Moutounet M. (2002). Aggregation of grape seed tannins in model wine—effect of wine polysaccharides. Food Hydrocoll..

[51] Springer L.F., Chen L.A., Stahlecker A.C., Cousins P., Sacks G.L. (2016). Relationship of soluble grape-derived proteins to condensed tannin extractability during red wine fermentation. J. Agric. Food Chem..

[52] Springer L.F., Sherwood R.W., Sacks G.L. (2016). Pathogenesis-related proteins limit the retention of condensed tannin additions to red wines. J. Agric. Food Chem..

[53] Watrelot A.A., Schulz D.L., Kennedy J.A. (2017). Wine polysaccharides influence tannin-protein interactions. Food Hydrocoll..

[54] Taira S., Ono M., Matsumoto N. (1997). Reduction of persimmon astringency by complex formation between pectin and tannins. Postharvest Biol. Technol..

[55] Carvalho E., Póvoas M.J., Mateus N., De Freitas V. (2006). Application of flow nephelometry to the analysis of the influence of carbohydrates on protein–tannin interactions. J. Sci. Food Agric..

[56] Mitropoulou A., Hatzidimitriou E., Paraskevopoulou A. (2011). Aroma release of a model wine solution as influenced by the presence of non-volatile components. Effect of commercial tannin extracts, polysaccharides and artificial saliva. Food Res. Int..

[57] Saenz-Navajas P., Tao Y., Dizy M., Ferreira V., Fernández-Zurbano P. (2010). Relationship between Nonvolatile Composition and Sensory Properties of Premium Spanish Red Wines and Their Correlation to Quality Perception. J. Agric. Food Chem..

